# Three-Dimensional Porous Artemia Cyst Shell Biochar-Supported Iron Oxide Nanoparticles for Efficient Removal of Chromium from Wastewater

**DOI:** 10.3390/molecules30081743

**Published:** 2025-04-13

**Authors:** Yu Gao, Ying Liu, Xu Zhao, Xinchao Liu, Qina Sun, Tifeng Jiao

**Affiliations:** State Key Laboratory of Metastable Materials Science and Technology, Hebei Key Laboratory of Heavy Metal Deep-Remediation in Water and Resource Reuse, School of Environmental and Chemical Engineering, Yanshan University, Qinhuangdao 066004, China; gaoyu0215@stumail.ysu.edu.cn (Y.G.); ying.liu@stumail.ysu.edu.cn (Y.L.); zhaoxu19981201@163.com (X.Z.); lance.simon@foxmail.com (X.L.)

**Keywords:** three-dimensional hierarchical porous biochar, *Artemia* cyst shell, hexavalent chromium (Cr(VI)), synergistic adsorption-reduction

## Abstract

Chromium-containing wastewater poses severe threats to ecosystems and human health due to the high toxicity of hexavalent chromium (Cr(VI)). Although iron oxide nanoparticles (IONPs) show promise for Cr(VI) removal, their practical application is hindered by challenges in recovery and reuse. Herein, a novel three-dimensional porous nanocomposite, *Artemia* cyst shell biochar-supported iron oxide nanoparticles (ACSC@ IONP), was synthesized via synchronous pyrolysis of Fe^3+^-impregnated *Artemia* cyst shells (ACSs) and in situ reduction of iron. The optimized composite C@Fe-3, prepared with 1 mol/L Fe^3+^ and pyrolyzed at 450 °C for 5 h, exhibited rapid removal equilibrium within 5–10 min for both Cr(VI) and total chromium (Cr(total)), attributed to synergistic reduction of Cr(VI) to Cr(III) and adsorption of Cr(VI) and Cr(III). The maximum Cr(total) adsorption capacity was 110.1 mg/g at pH 2, as determined by the Sips isothermal model for heterogeneous adsorption. Competitive experiments demonstrated robust selectivity for Cr(VI) removal even under a 64-fold excess of competing anions, with an interference order of SO_4_^2−^ > NO_3_^−^ > Cl^−^. Remarkably, C@Fe-3 retained 65% Cr(VI) removal efficiency after four adsorption–desorption cycles. This study provides a scalable and eco-friendly strategy for fabricating reusable adsorbents with dual functionality for chromium remediation.

## 1. Introduction

Chromium (Cr), an essential industrial resource, plays a crucial role in alloy production, aerospace materials, and fireproofing. The discharge of Cr-containing wastewater poses significant environmental and health risks, primarily due to the high toxicity and mobility of hexavalent chromium (Cr(VI)) [1,2,3]. Compared to trivalent chromium (Cr(III)), which is less toxic and plays a vital role in metabolic processes, Cr(VI) exhibits greater oxidative potential and environmental persistence, making it a critical target for wastewater treatment [4,5,6]. Although Cr(III) is generally considered to be less toxic, it may be converted into more toxic Cr(VI) in the presence of strong oxidants such as free chlorine and manganese oxides, thereby increasing its environmental and health risks [7,8,9,10]. This transformation underscores the importance of addressing both Cr(III) and Cr(VI) in total chromium (Cr(total)) purification. Consequently, Cr-containing wastewater, with its widespread sources and severe environmental impacts, has become a major concern in heavy metal remediation. Therefore, developing efficient and sustainable treatment methods for Cr(total) removal is of paramount importance.

Adsorption has emerged as a promising method among the various Cr-containing wastewater treatment technologies due to its ability to achieve lower residual chromium concentrations, even at low initial concentrations [11,12,13,14]. Among adsorbents, nanomaterials have been the most extensively studied for their high surface area, tunable surface chemistry, and exceptional adsorption capacity [15,16]. Iron oxide nanoparticles (IONPs) exhibit exceptional Cr(VI) removal efficiency. Tumutungire et al. [17] synthesized IONPs from steel pickling iron oxide waste, demonstrating a remarkable Cr removal efficiency of 99% in both synthetic and industrial wastewater. Chatterjee et al. [18] showed that using NaOH solution could effectively elute Cr(VI) adsorbed by iron oxide nanoparticles, thus achieving material regeneration. At the same time, their experimental data show that after five consecutive cycles of adsorption and elution, IONPs can still maintain the removal efficiency of Cr(VI) at about 80%, which proves that IONPs have excellent recycling performance in practical applications. However, IONPs tend to aggregate during water treatment, leading to a loss of nanoscale activity. Aggregated IONPs not only hinder separation from treated water in static reactors, affecting effluent quality, but also cause clogging in dynamic adsorption reactors, impairing their operation.

To address these challenges, significant research efforts have focused on achieving uniform dispersion and maintaining the nanoscale activity of nanoparticles. A promising solution lies in the use of natural porous biomass; however, most conventional plant biomass (e.g., lotus stems [19], rice husks [20], macroalgae [21]) exhibit monodisperse micropores that restrict nanoparticle dispersion and cause severe mass transfer limitations. In contrast, *Artemia* cyst shells (ACSs)—featuring a unique three-dimensional (3D) hierarchical porous structure—offer a novel strategy to overcome these constraints. The inner layer of ACSs contains numerous nanopores, providing a microporous template for nanoparticle anchoring, while the outer macroporous layer enhances aqueous-phase mass transfer [22,23]. ACSs and ACS-based composites have been widely explored for pollutant removal [23,24,25,26,27]. Wang et al. investigated ACSs for heavy metal cation adsorption [23] and modified ACSs with TiO_2_ for formaldehyde degradation [24]. ACS-supported nano-Mg(OH)_2_ composites were developed for phosphate removal [25], while ACS-loaded nano-Ag demonstrated antibacterial properties [26]. The 3D porous structure of ACSs was utilized to achieve rapid lead ion adsorption [27], and copper-modified ACS composites effectively removed iodide and iodate anions [28]. Despite these advances, few studies have leveraged the hierarchical porosity of ACSs to simultaneously provide adsorption and redox reaction sites. This dual functionality is crucial for the effective removal of Cr(total). Carbonization offers a promising solution for this need, as it can generate biochar with abundant surface functional groups and favorable redox conditions [28,29], enhancing the ability to remove both Cr(VI) and Cr(III).

In this work, the in situ formation and uniform distribution of IONPs in the three-dimensional porous network of *Artemia* cyst shell biochar (ACSC) were achieved by simultaneous pyrolysis and reduction. The composite material is a new type of carbon-based nanocomposite with both adsorption and redox capabilities. It not only exhibits high nanoscale activity, but also shows practical application potential in engineering applications. The composite was characterized by XRD, Raman spectroscopy, FTIR, SEM, and TEM, and its Cr adsorption performance was evaluated under varying pHs, competitive ions, isothermal adsorption, and kinetic conditions. In the regeneration experiment, NaOH and HCl solutions were used as desorption agents to prove its practical applicability. Mechanistic studies revealed that the dual adsorption and redox capabilities of ACSC@IONP enabled the selective removal of both Cr(VI) and total chromium, providing an efficient solution for chromium-containing wastewater treatment.

## 2. Results and Discussion

### 2.1. Characterization

**Figure 1:**
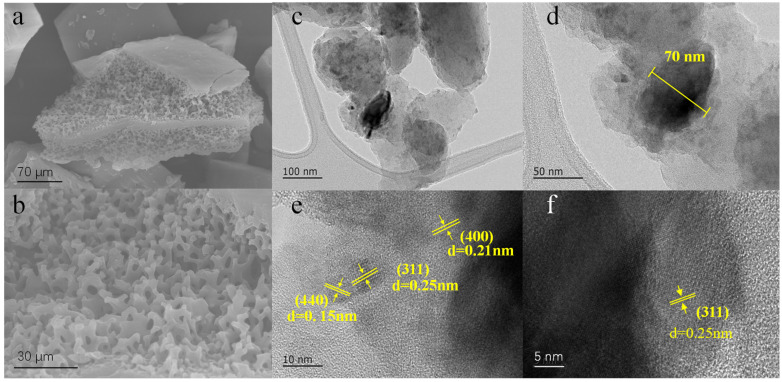
The characterizations of ACSC@IONP: (**a**,**b**) the SEM micrographs of C@Fe-3; (**c**–**f**) the TEM micrographs of C@Fe-3.

The microstructure of the ACSC@IONP was investigated using scanning electron microscopy (SEM) and transmission electron microscopy (TEM). As shown in SEM images in Figure 1a,b, the typical ACSC@IONP composite C@Fe-3 inherited a sparse, hierarchical porous structure from the original *Artemia* cyst shell (ACS) [23], which facilitates pollutant adsorption. Elemental microregion analysis (SEM-EDS, Figure S1 and Table S1) confirmed the presence of oxygen (O), iron (Fe), and carbon (C), indicating that iron oxide nanoparticles had been successfully loaded onto the biochar, and the loading of Fe was 21.2 wt.%. High-resolution transmission electron microscopy (TEM) images provided direct evidence of crystalline iron oxide nanoparticles anchored to the ACS surfaces (Figure 1c–f). Diffraction fringes corresponding to the (311), (440), and (400) lattice planes of iron oxide nanoparticles were observed, with interplanar spacings of 0.25 nm, 0.15 nm, and 0.21 nm, respectively. The iron oxide nanoparticles particles exhibited an average size of ~70 nm, confirming successful crystallization and dispersion.

A Brunauer–Emmett–Teller (BET) specific surface area analyzer was used to evaluate the specific surface area, pore size and pore volume of ACSCs and ACSC@IONP. Figure S2a shows the nitrogen adsorption–desorption isotherms of these materials in a nitrogen atmosphere. According to the classification standard of isotherms, the adsorption isotherms of ACSC, C@Fe-1, C@Fe-2 and C@Fe-3 show Type IV isotherms, which indicates that they have well-developed mesoporous structures [30]. Analysis of pore size distribution (Figure S2b) further confirmed the presence of mesopores and macropores in ACSC, C@Fe-1, C@Fe-2 and C@Fe-3. In addition, the data in Table S2 show that the specific surface area of the material can be increased by properly introducing FeCl_3_ solution and prolonging the carbonization time. When the pyrolysis temperature was increased to 600 °C, C@Fe-4 experienced complete carbonization, resulting in the loss of its pore structure. Therefore, it can be inferred that the enhanced surface area and porous structure of C@Fe-3 may increase the number of active sites of C@Fe-3 and its effective contact area with Cr(VI), resulting in relatively high adsorption efficiency [31].

**Figure 2:**
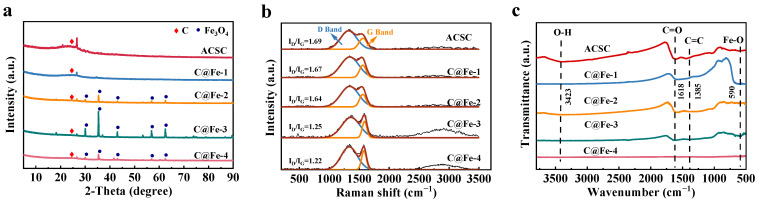
(**a**) XRD patterns, (**b**) Raman spectra, and (**c**) FTIR spectra of ACSC and ACSC@IONP.

The phases of ACSC and the four ACSC@IONP composites were analyzed using X-ray diffraction (XRD), as shown in Figure 2a. ACSC exhibited a broad peak at 2*θ* = 23°, corresponding to the (002) plane of amorphous carbon, confirming the amorphous nature of the ACS-derived biochar [32]. Among the four ACSC@IONP composites, the SEM-EDS (Figure S3) of C@Fe-1 (prepared with 0.5 mol/L Fe^3+^ at 450 °C for 3.5 h) showed O and Fe peaks, indicating that iron oxides were successfully generated on its surface. However, the XRD image did not show crystalline Fe_3_O_4_ peaks, indicating inadequate conditions for Fe_3_O_4_ crystallization. In contrast, C@Fe-2, C@Fe-3, and C@Fe-4 displayed distinct diffraction peaks at 2*θ* = 30.04°, 35.38°, 43.04°, 56.09°, and 62.48°, corresponding to the (220), (311), (400), (511), and (440) planes of Fe_3_O_4_, respectively (Fe_3_O_4_: PDF#99-0073) [33]. This demonstrated that increasing Fe^3^⁺ concentration, pyrolysis temperature, or duration promoted Fe_3_O_4_ crystallization. However, Fe_3_O_4_ sizes varied significantly: 109 nm for C@Fe-2, 76.9 nm for C@Fe-3, and 59.9 nm for C@Fe-4, reflecting the influence of preparation conditions. Notably, C@Fe-3 (1.0 mol/L Fe^3+^, 450 °C, 5 h) exhibited the highest peak intensity, indicating optimal crystallinity among the composites.

Raman spectroscopy (Figure 2b) further elucidated the structural properties of the composites. All four ACSC@IONP composites displayed characteristic D and G bands at ~1340 cm^−1^ and ~1580 cm^−1^, respectively, representing disordered carbon structures (D band) and graphitic carbon (G band). The intensity ratio I_D_/I_G_ was used to assess the graphitization degree. The I_D_/I_G_ values for ACSC, C@Fe-1, C@Fe-2, C@Fe-3, and C@Fe-4 were 1.69, 1.67, 1.64, 1.25, and 1.22, respectively. The D peak arises from disordered structures or defects within the biochar lattice, whereas the G peak is associated with the vibration of sp^2^-hybridized carbon atoms and the aromatic ring system in the biochar [34]. Additionally, the G band is considered to be the result of the E_2g_ symmetric vibration mode in an ideal graphite lattice, with its position reflecting the degree of charge transfer [35,36,37]. Thus, a lower I_D_/I_G_ ratio indicates a higher degree of graphitization with C@Fe-4 showing the highest graphitization due to complete carbonization at 600 °C. The reduced I_D_/I_G_ values for C@Fe-2, C@Fe-3, and C@Fe-4 compared to C@Fe-1 suggested that longer pyrolysis time, higher FeCl_3_ concentration, and elevated temperature facilitated the transformation of amorphous carbon into graphitic structures, enhancing electron transfer and Cr(VI) reduction efficiency.

Fourier-transform infrared (FTIR) spectroscopy (Figure 2c) revealed changes in functional groups after pyrolysis. For C@Fe-4, functional groups nearly disappeared, consistent with complete carbonization at high temperature, as supported by Raman analysis. In C@Fe-3, a new peak appeared at 590 cm^−1^, corresponding to the stretching vibration of Fe-O, indicating the presence of Fe_3_O_4_ particles [33]. For C@Fe-1, C@Fe-2, and C@Fe-3, a peak at 1618 cm^−1^ was attributed to C=O stretching in carboxyl groups -COOH, which can serve as adsorption sites for Cr ions through complexation. A peak at 1385 cm^−1^ was assigned to C=C stretching vibrations [38]. Variations in peak intensities between the composites further demonstrated that Fe^3+^ concentration, pyrolysis temperature, and duration significantly influenced the chemical properties of surface functional groups.

These structural and compositional results highlight the promising advantages of C@Fe-3: (i) a porous network inherited from ACS for enhanced mass transfer and adsorption capacity, and (ii) well-dispersed Fe_3_O_4_ nanoparticles that generate carbon-centered radicals and graphite-like domains, and (iii) a graphitic biochar matrix offering additional redox-active sites, synergistically enabling efficient Cr(VI) reduction and Cr(total) immobilization [39,40]. The composite’s ability to adsorb chromium ions was preliminarily demonstrated by distinct Cr peaks in the post-adsorption EDS analysis (Figure S1).

### 2.2. Chromium Removal Efficiency of ACSC@IONP

**Figure 3:**
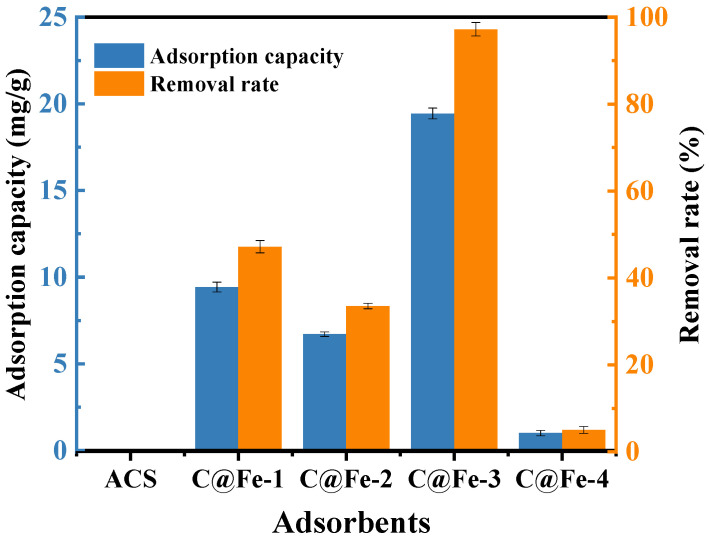
The adsorption capacity and removal rate of Cr(VI) onto different adsorbents, adsorbent dose 25 mg/50 mL, initial concentration of Cr(VI) 10 mg/L, 25 °C, 24 h, pH = 2.

Figure 3 compares the Cr(VI) adsorption capacities of four ACSC@IONP composites under identical experimental conditions, using ACSC as the reference material. ACSC alone exhibited negligible Cr(VI) removal efficiency, whereas the composite C@Fe-3 achieved a remarkable removal efficiency of 97.2%, highlighting the critical role of IONPs in Cr(VI) immobilization. Notably, C@Fe-3 outperformed other ACSC@IONP composites (C@Fe-1, C@Fe-2, and C@Fe-4) and raw ACS. This superior performance is attributed to the synergistic effects of Fe_3_O_4_ crystallization and the hierarchical porous structure of ACSC, which collectively enhance adsorption and redox activity.

**Figure 4:**
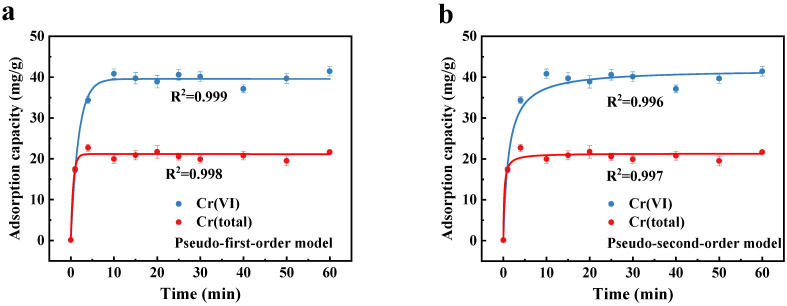
Removal kinetics of Cr(VI) and Cr(total) on C@Fe-3 fitted with (**a**) the pseudo-first-order model and (**b**) the pseudo-second-order model, C@Fe-3 dose 100 mg/L, initial concentration of Cr(VI) 50 mg/L, pH = 2.

The kinetics data of Cr(VI) and Cr(total) removal by C@Fe-3 are presented in Figure 4. Cr(total) removal reached equilibrium within 5 min, while Cr(VI) removal required 10 min, reflecting a dual mechanism: (1) rapid adsorption of Cr(VI) via surface hydroxyl groups on Fe_3_O_4_ [41,42], and (2) subsequent reduction of adsorbed Cr(VI) to Cr(III) by Fe(II) in Fe_3_O_4_ crystal and π electrons provided by the graphitized biochar matrix [43,44,45]. The hierarchical porosity of ACSC is conducive to the rapid mass transfer of Cr(VI) to the adsorption site. In the first 5 min, Cr(VI) is rapidly reduced after being adsorbed by C@Fe-3 to form Cr(III), which is fixed on the surface of the material. Beyond this phase, the reduction process dominates, which is mainly driven by Fe(II) in Fe_3_O_4_ crystal and π-electrons of porous carbon. C@Fe-3 continued to adsorb Cr(VI), but the adsorption sites gradually became saturated, Cr(III) began to fall off from the surface of the material, and the system finally reached a dynamic equilibrium state. This electron transfer was further enhanced by the optimized electronic structure and spatial configuration of C@Fe-3, as evidenced by its high graphitization (low I_D_/I_G_ ratio) and stable Fe-O bonding [46]. The 3D pore hierarchy of ACSC@IONP addresses a critical limitation of Fe-loaded biochars—trade-offs between porosity and accessible sites. Specifically, this hierarchical porosity enables exceptional adsorption kinetics, a highest 112-fold rate enhancement over non-hierarchical Fe-biochar requiring 1.5–20 h for equilibrium (Table 1). This disparity originates from fundamental structural differences. The 3D hierarchal pores of ACSC provide (i) macropores acting as low-resistance pathways, shortening the diffusion distance compared to tortuous microporous networks; (ii) mesoporous walls densely decorated with IONPs, ensuring high accessibility of redox-active sites. Such structural advantages enable Cr(VI) to rapidly reach adsorption–reduction interfaces, and showed the rapid removal kinetics in the experiment. The pseudo-first-order and pseudo-second-order kinetic models were used to fit the adsorption data. The corresponding equations are shown in Equations (1) and (2), respectively, and the fitting results are summarized in Table S3. The experimental kinetic pseudo-first-order rate constant *K*_1_ = 0.555 min^−1^ for Cr(VI) adsorption, as detailed in Table S3, further corroborates this hierarchical pore-engineered acceleration.

Pseudo-first-order model,

(1) $$Q_{t}=Q_{e}(1-e^{{-K}_{1}t})$$

Pseudo-second-order model,

(2) $$\frac{t}{Q_{t}}=\frac{1}{K_{2}Q_{e}^{2}}+\frac{t}{Q_{e}}$$

Here, *K*_1_ (1/min) and *K*_2_ (g/(mg∙min)) denote the pseudo-first-order and pseudo-second-order constants, respectively.

### 2.3. Isothermal Adsorption

Classical Langmuir, Freundlich and Sips models were used to fit the isothermal adsorption experimental data. The Langmuir model is derived from the ideal assumptions of monolayer adsorption, uniform adsorbent surface, dynamic adsorption equilibrium and no interaction between adsorbed molecules, which can better describe the uniform adsorption process. The model is expressed as Equation (3) [57,58]. The Freundlich model was first proposed to describe irrational and reversible adsorption reactions. The model is suitable for multilayer adsorption with heterogeneous surface and heterogeneous adsorption heat and affinity. The model is expressed as Equation (4), where *n* is a measure of adsorption strength or surface heterogeneity, *n* > 1 means a chemical adsorption process, and *n* < 1 means synergistic adsorption [57,58]. The defect of this model lies in the support of thermodynamic theory; it does not conform to Henry’s law at low concentration. Sips model is a combination of the Langmuir model and Freundlich model. The Sips isothermal model represents the monolayer adsorption of one adsorbate molecule onto 1/*n*_s_ adsorption sites in the heterogeneous systems. The model is expressed as Equation (5), where *K*_s_ is related to adsorption affinity [57,58].

(3) $$q_{e}=\frac{Q_{max}bC_{e}}{1+bC_{e}}$$

where *q*_e_ is the adsorption capacity of the adsorbent (mg/g), *C*_e_ is the concentration of Cr(VI) or Cr(total) at equilibrium (mg/L), *Q*_max_ is the maximum adsorption capacity (mg/g), *b* is the Langmuir constant (L/mg).

(4) $$q_{e}=K_{F}C_{e}^{\frac{1}{n}}$$

where *K*_F_ is Freundlich coefficient (mg/g), *n* is Freundlich coefficient.

(5) $$q_{e}=Q_{max}\frac{{\left(K_{s}C_{e}\right)}^{n_{s}}}{1+{\left(K_{s}C_{e}\right)}^{n_{s}}}$$

where *K*_s_ is the reaction equilibrium constant (L·mg^−1^), *n*_s_ is the heterogeneity coefficient.

**Figure 5:**
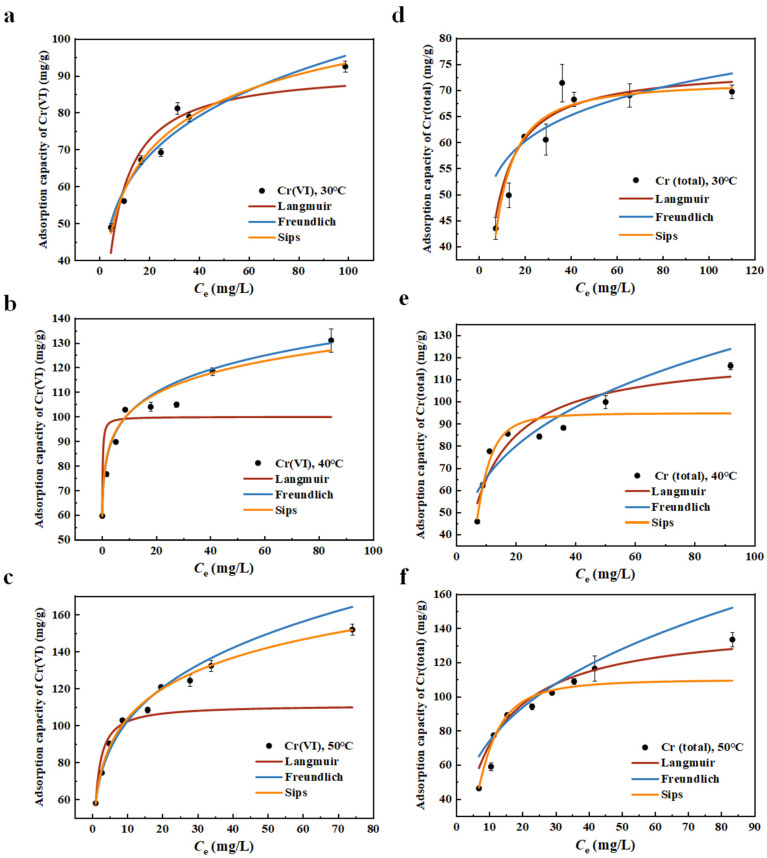
The isothermal adsorption data of (**a**–**c**) Cr(VI) and (**d**–**f**) Cr(total) on C@Fe-3 at 30 °C, 40 °C, and 50 °C, respectively; C@Fe-3 dose 25 mg/50 mL, initial concentration of Cr(VI) 20~100 mg/L, 24 h, pH 2.

The fitting results of the three isothermal adsorption models to the adsorptions’ data of Cr(VI) and Cr(total) are illustrated in Figure 5, and the regression coefficients are listed in Table S4. According to the regression coefficients, the isothermal data had good consistenccy with the Sips isothermal model, which indicated that the heterogeneous adsorption system of Cr(VI) and Cr(total) on the C@Fe-3 surface and the one adsorbate molecule onto 1/*n*_s_ adsorption sites during the removal of Cr(VI) and Cr(total). According to the Langmuir and Freundlich models, the adsorptions were endothermic chemical adsorption, and the adsorption capacities increased with the temperature increase. The maximum adsorption capacity of Cr(total) on C@Fe-3 at pH 2 was 110.1 mg/g.

### 2.4. pH-Dependent Cr(VI) Removal

**Figure 6:**
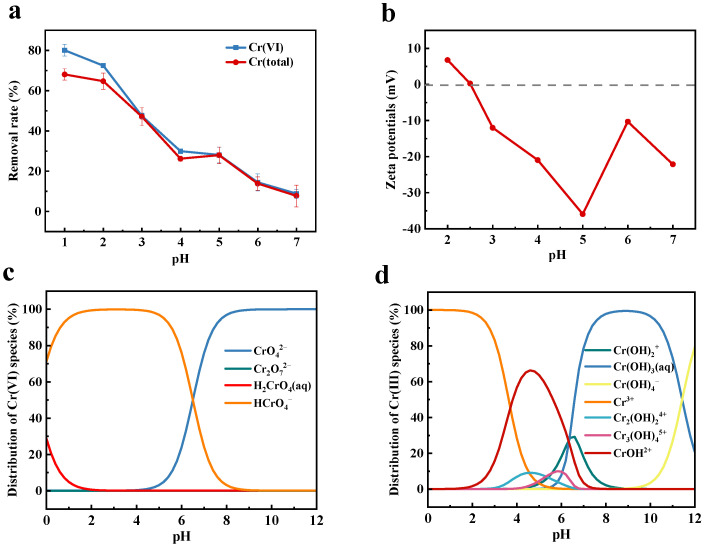
(**a**) Removal rate of Cr(VI) and Cr(total) at different pH levels, C@Fe-3 dose 25 mg/50 mL, initial concentration of Cr(VI) 50 mg/L, 24 h, 25 °C; (**b**) zeta potentials of C@Fe-3; (**c**,**d**) the speciation diagram for Cr(VI) and Cr(III) in aqueous solution as a function of pH.

Figure 6a illustrates the pH-dependent removal efficiency of Cr(VI) and Cr(total) by C@Fe-3. Both Cr(VI) and Cr(total) removal rates exhibited a three-stage decline as pH increased from 1 to 7. At pH < 2, maximum removal efficiencies of 80.0% for Cr(VI) and 68.1% for Cr(total) were achieved at pH = 1, attributed to strong electrostatic attraction between the positively charged C@Fe-3 surface and the predominant Cr(VI) anions HCr_2_O_7_^−^. In the range of 2 < pH < 5, removal rates dropped sharply to ~28% at pH 5, while further reduction occurred at pH > 5. This trend is governed by three interrelated factors that collectively dictated chromium removal efficiency: surface charge modulation, competitive adsorption, and chromium speciation. Surface charge modulation played a pivotal role, as evidenced by zeta potential measurements (Figure 6b) showing an isoelectric point at pH 2.5. Below this critical pH, the composite material is positively charged and attracts Cr(VI) oxygen anions; in contrast, it is negatively charged, resulting in electrostatic repulsion. The observed zeta potential increase within 5 < pH < 6 is likely due to deprotonation of hydroxyl/phenolic groups on the biochar surface under alkaline conditions [59]. This charge transition synergized with competitive adsorption dynamics, where escalating OH^−^ concentrations at elevated pH values progressively occupied available adsorption sites, thereby diminishing Cr(VI) removal capacity. Concurrently, chromium speciation also affects the adsorption process, as the dominant CrO_4_^2−^ species (pH > 5) required more adsorption-free energy compared to the HCrO_4_^−^ form prevalent in acidic media [60], further reducing the removal rate. It is also noteworthy that the removal rate of Cr(VI) was higher than that of Cr(total) at pH ≤ 2, while their efficiencies converged at pH 3–7. This is due to the partial reduction of Cr(VI) to soluble Cr(III). At pH < 2, C @ Fe-3 is positively charged, and electrostatic attraction adsorbs HCrO_4_^−^, so that Cr(III) remains in the solution. With increasing OH^−^ concentration, C@Fe-3 is negatively charged, and Cr(III) is almost completely adsorbed, resulting in a consistent removal rate of Cr(VI) and Cr(total) at pH 3~7.

### 2.5. Selective Cr(VI) Removal in the Presence of Competing Anions

**Figure 7:**
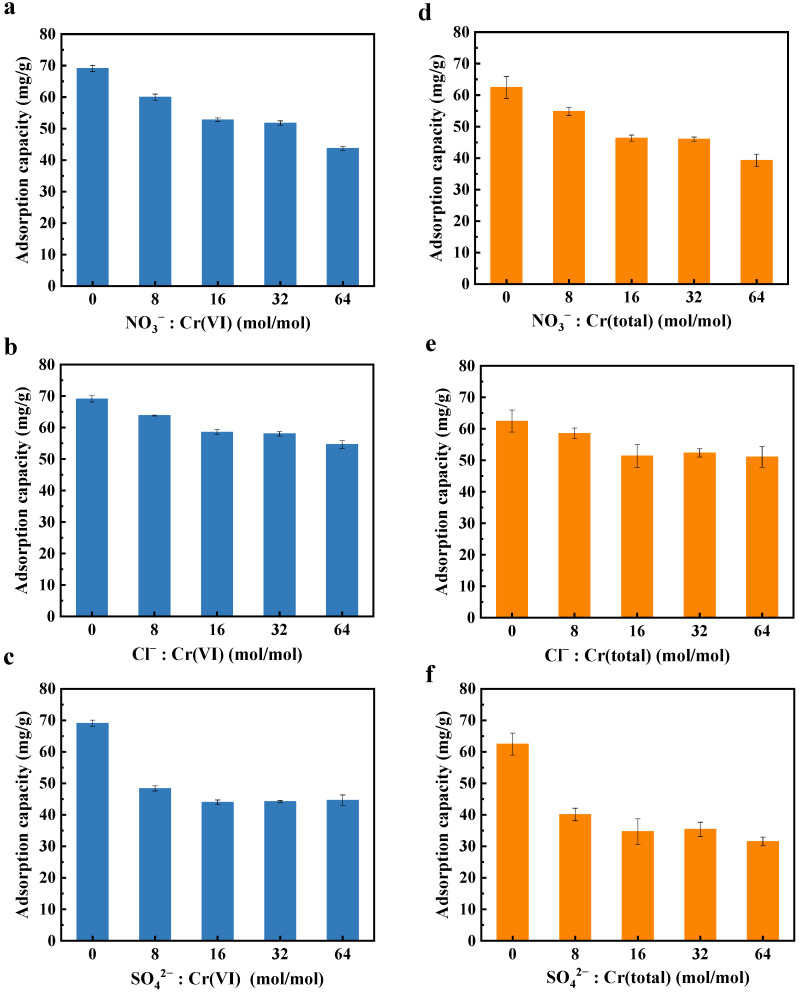
The effects of NO_3_^−^, Cl^−^ and SO_4_^2−^ on the adsorption of Cr(VI) (**a**–**c**) and Cr(total) (**d**–**f**) by C@Fe-3, C@Fe-3 dose 25 mg/50 mL, initial concentration of Cr(VI) 50 mg/L, 24 h, pH = 2.

The effects of common anions (NO_3_^−^, Cl^−^, and SO_4_^2−^) on the removal of Cr(VI) and Cr(total) by the C@Fe-3 composite were investigated (Figure 7). All three anions reduced Cr(VI) adsorption capacity by approximately 20%, primarily due to competition with Cr(VI) oxyanions Cr_2_O_7_^2−^ and KCr_2_O^7−^ for surface adsorption sites. This competition weakened the adsorbent’s ability to enrich target anions, and since Cr(VI) adsorption precedes its reduction, Cr(total) removal efficiency also decreased accordingly. However, when competing anion concentrations reached 16-fold of Cr(VI) (mol/mol), the removal efficiency stabilized, demonstrating the composite’s robust performance even in high-ionic-strength environments.

The inhibitory effects followed the order: SO_4_^2−^ > NO_3_^−^ > Cl^−^ for both Cr(VI) and Cr(total). SO_4_^2−^, with the highest negative charge and molecular weight, exhibited the strongest electrostatic attraction to the positively charged C@Fe-3 surface at pH 2, thereby occupying more adsorption sites and exerting the greatest interference. This trend highlights the critical role of anion charge density and molecular size in competitive adsorption. Importantly, the composite retained significant chromium-removal efficiency despite high concentrations of competing ions, underscoring the selective adsorption capability conferred by the Fe_3_O_4_ nanocrystals and hierarchical porous structure of ACSC [61,62].

### 2.6. Reusability and Stability Evaluation

**Figure 8:**
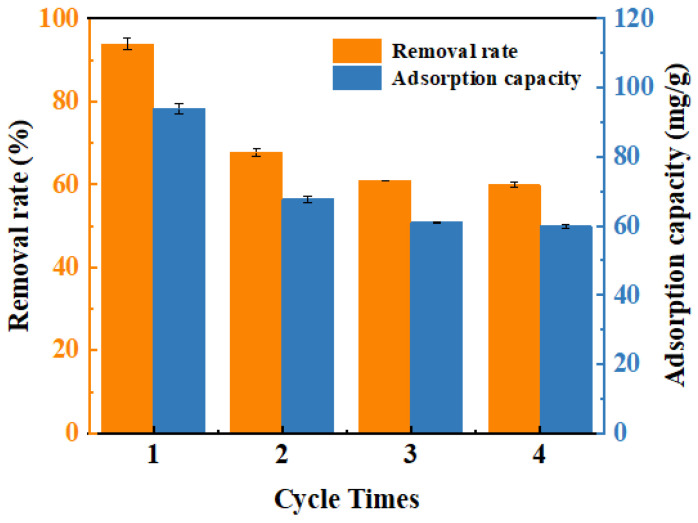
The removal rate and adsorption capacity of Cr(VI) by C@Fe-3 in the adsorption–desorption cycles.

The reusability of ACSC@INOP was systematically evaluated through sequential desorption–adsorption cycles (Figure 8). The regeneration experiments were carried out using 0.5 mol/L NaOH, 0.1 mol/L NaOH and 0.1 mol/L HCl, respectively. The data are shown in Figure S4. The use of 0.5 mol/L NaOH and 0.1 mol/L NaOH as desorption agents had similar desorption effects, and the desorption rate was significantly higher than that of 0.1 mol/L HCl. NaOH is a better eluent, which may be mainly due to the negative charge of C@Fe-3 under alkaline conditions, resulting in the desorption and release of chromium into the solution again. Similarly, the above findings may be another possible explanation for the orange-yellow color reappearance of the solution during desorption [63]. After regeneration with 0.1 mol/L NaOH, the content of Fe in C@Fe-3 decreased significantly (Table S1), which may be due to the partial dissolution of Fe_2_O_3_ nanoparticles during alkali treatment. Subsequent cycles 3 and 4 showed minimal further Fe loss, indicating that the residual Fe material was stabilized by a strong interaction with the biochar matrix. Although the leaching of iron occurred, the data of the leaching experiment showed that the leaching rate of iron was only 1.71% (Table S5), and the Cr(VI) removal efficiency was stable at about 65% in cycles 2–4, which was about 30% lower than that of the fresh adsorbent. This performance attenuation likely stems from irreversible structural alterations: NaOH treatment failed to restore the sp^2^ carbon framework of ACSC, resulting in the surface-bound Cr(VI) not being completely desorbed, thereby reducing the available active sites in the subsequent cycles. The retained stability in later cycles highlights the robustness of the remaining Fe_3_O_4_–biochar hybrid structure, where preserved porous channels and residual Fe-O-C linkages maintained partial adsorption-reduction functionality.

### 2.7. Mechanism of Chromium Removal

**Figure 9:**
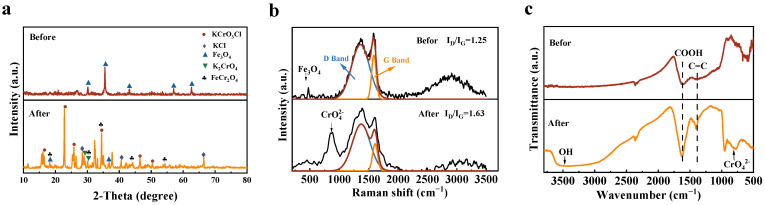
Characterization changes of C@Fe1-3 after chromium adsorption: (**a**) XRD patterns (Fe_3_O_4_: PDF#99-0073, KCl: PDF#73-0380, K_2_CrO_4_: PDF#70-1489, KCrO_3_Cl: PDF#74-0907); (**b**) Raman spectra; (**c**) FTIR spectra.

To elucidate the adsorption mechanism of chromium on ACSC@IONP, XRD, Raman, and FTIR analyses were conducted before and after adsorption (Figure 9). XRD patterns revealed significant phase and structural changes post-adsorption: the characteristic (311) crystal plane of Fe_3_O_4_ nearly vanished, while residual (200) and (400) planes persisted, indicating preferential involvement of the (311) facet in chromium binding. New phases, including FeCr_2_O_4_, KCl, and K_2_CrO_4_, were identified, confirming Cr(VI) reduction to Cr(III) and subsequent Fe-Cr co-precipitation on the composite surface. Raman spectroscopy further supported this mechanism, where the appearance of CrO_4_^2−^ peaks and an elevated I_D_/I_G_ ratio of 1.63 suggested π-electron transfer from sp^2^-hybridized carbon to reduce Cr(VI), which disrupted the graphitic structure. FTIR analysis showed enhanced peaks for -OH, C=C, and -COOH groups after adsorption, implicating these protonatable oxygen-containing functionalities in Cr(VI) oxyanion capture through electrostatic interactions under acidic conditions.

**Figure 10:**
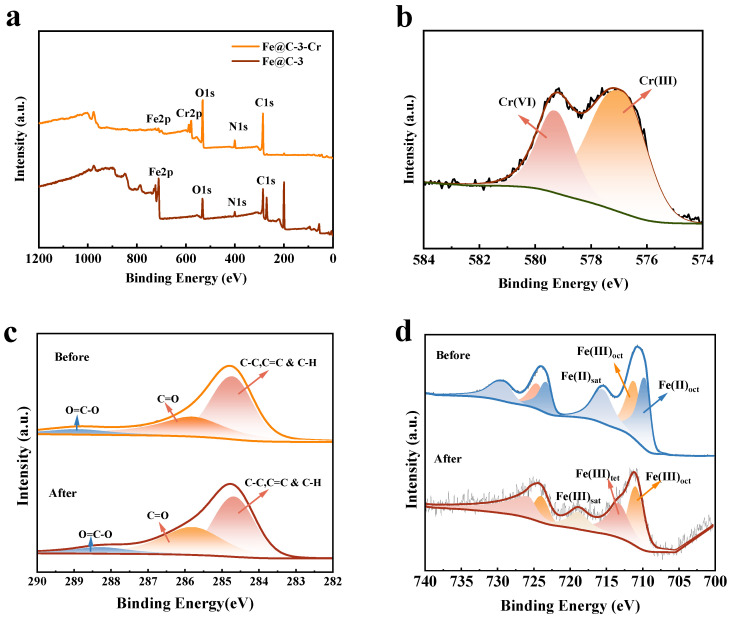
XPS spectra of C@Fe-3 before and after Cr(VI) adsorption: (**a**) survey spectra; (**b**) Cr 2p3/2; (**c**) C 1s; (**d**) Fe 2p3/2.

Figure 10a shows that there is a clear chromium peak on the surface of C@Fe-3 after adsorbing Cr(VI), which confirms the successful adsorption of pollutants. Further XPS analysis (Figure 10b) showed that the adsorbed C@Fe-3 had binding energy peaks at 577.01 eV and 579.24 eV, which were attributed to Cr(III) and Cr(VI) [33], accounting for 61.78% and 38.22%, respectively. This result indicates that most of the Cr(VI) adsorbed on the surface of C@Fe-3 is reduced to Cr(III) during the reaction. After the adsorption of Cr(VI), the C1s peak of C@Fe-3 was decomposed into three main components (Figure 10c), and the binding energies were located at 284.68 eV, 284.80 eV and 288.24 eV, respectively, corresponding to C-C, C=O and O=C-O [64]. Among them, the proportion of C-C decreased, while the proportion of C=O and O=C-O increased slightly. This indicates that during the Cr(VI) reduction process, the carbon layer of C@Fe-3 undergoes an oxidation reaction, generating more carbonyl (C=O) and carboxyl (O=C-O) functional groups. These new functional groups may provide binding sites for the reduced Cr(III). The Fe 2p3/2 spectrum of C@Fe-3 (Figure 10d) can be decomposed into three peaks with binding energies of 709.74 eV, 711.25 eV and 724.58 eV, which are attributed to Fe(III) in octahedral coordination (Fe(III)oct), Fe(III) in tetrahedral coordination (Fe(III)tet) and Fe(II) in octahedral coordination (Fe(II)oct) in Fe_3_O_4_, respectively [34]. After the adsorption and reduction of Cr(VI), the Fe(II)oct peak almost completely disappeared, and a new satellite peak belonging to Fe(III) appeared at 717.70 eV. This indicates that electron transfer occurs during the reaction: Fe(II) is oxidized to Fe(III), while Cr(VI) is reduced to Cr(III).

**Figure 11:**
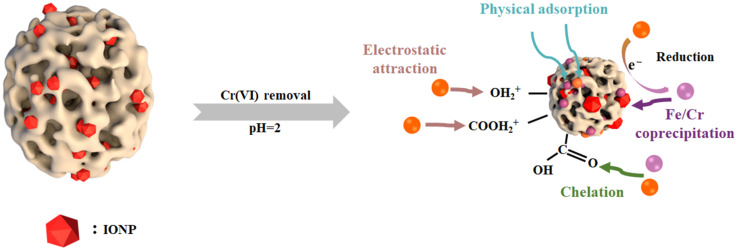
A proposed mechanism of Cr adsorption by the ACSC@IONP nanocomposite.

The proposed chromium removal mechanism by ACSC@IONP involves four pathways (Figure 11): (i) electrostatic attraction of Cr(VI) oxyanions to the positively charged ACSC@IONP surface; (ii) partial Cr(VI) immobilization via surface functional group complexation; (iii) π-electrons from the carbon matrix and Fe(II) act as electron donors, reducing Cr(VI) to Cr(III); (iv) the reduced Cr(III) either precipitates as FeCr_2_O_4_ on the composite or is re-adsorbed via functional groups, while excess Cr(III) remains in the water due to electrostatic repulsion at a lower pH value. The hierarchical 3D porous structure of ACSC enhances mass transfer and nanoparticle dispersion, synergistically enabling efficient chromium sequestration through coupled adsorption-reduction processes.

## 3. Materials and Methods

### 3.1. Materials

Potassium dichromate (K_2_Cr_2_O_7_) was purchased from Northern Chemical Glass Purchase and Sales Center (Tianjin, China). NaOH was purchased from Aladdin Biochemical Technology Co., Ltd., Shanghai, China. H_3_PO_4_ (85%), H_2_SO_4_ (95 wt%–98 wt%), HCl (37 wt%), absolute ethanol, and diphenylcarbazide were purchased from Kaitong Chemical Reagent Co., Ltd. (Tianjin, China). Na_2_SO_4_, NaNO_3_, NaCl, and FeCl_3_ were purchased from Kemiou Chemical Reagent Co., Ltd. (Tianjin, China). ACSs were obtained from Qinhuangdao Fisheries Research Institute (Qinhuangdao, China). All chemicals are analytical grade and were used without further purification.

### 3.2. Preparation of Artemia Cyst Shell Biochar-Supported Iron Oxidenanoparticles ACSC@IONP

Pretreatment of ACSs: 10 g of original ACSs was added to 300 mL ethanol–water solution (50 wt%) by stirring for 5 h to remove salt and residual impurities. This step was repeated 3 to 4 times. Then, the washed ACSs were put in an ultrasonic cleaner to rinse for 30 min until the ACSs precipitated to the bottom of the water so that the internal pores were entirely penetrated by water. Then the rinsed ACSs were dried at 60 °C for 24 h.

**Figure 12:**
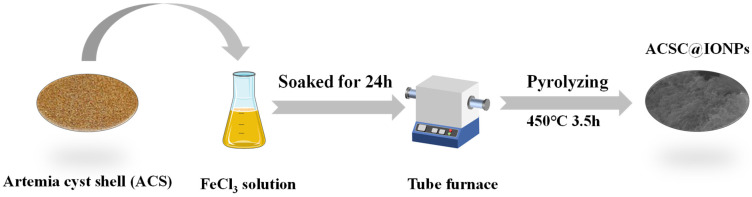
Preparation schematic of ACSC@IONP.

Preparation of ACSC@IONP: 5 g of rinsed ACSs was immersed in a 250 mL 0.5 mol/L FeCl_3_ solution and stirred for 4 h, filtered and dried at 60 °C for 8 h. Then, the obtained solids were pyrolyzed at 450 °C for 3.5 h, with a heating rate of 12.5 °C/min, in a tube furnace under nitrogen protection. The prepared ACSC@IONP was denoted as C@Fe-1. Three more ACSC@IONP were prepared at different Fe^3+^ concentrations, pyrolysis temperatures and times, and denoted as C@Fe-2 (0.5 mol/L FeCl_3_, 450 °C, 5 h), C@Fe-3 (1 mol/L FeCl_3_, 450 °C, 5 h), and C@Fe-4 (0.5 mol/L FeCl_3_, 600 °C, 3.5 h), respectively. The ACSC was prepared for comparison by the same pyrolysis but without immersion. The preparation of the 4 ACSC@IONP samples is illustrated in Figure 12.

### 3.3. Characterizations

ACSC@IONP and ACSC samples were characterized by XRD, Raman, FTIR, SEM, and TEM. The crystal morphology of composites was characterized by X-ray diffraction (XRD, D/max 2550PC, Rigaku, Tokyo, Japan) with Cu Kα radiation (*λ* = 0.1542 nm) in the 2*θ* range of 5–90° at a scanning rate of 5° 2*θ*·min^−1^. The electronic structure information of samples was characterized by Raman spectroscopy (Lab RAM HR800, Horiba Jobin-Yvon, Paris, France). Surface functional groups were identified using FTIR (Nicolet iS10, Thermo Scientific, Billerica, MA, USA) in attenuated total reflectance mode (400–4000 cm^−1^). The microstructure and surface elements were analyzed by SEM (S-3400N II, Hitachi, Tokyo, Japan) coupled with EDS. The diffraction fringes and sizes of Fe_3_O_4_ crystal were observed by high-resolution TEM (JEM-2010FX, JEOL, Tokyo, Japan). The Zeta potential was analyzed by Zeta Potentiometer (Nano-ZS90, Malvern Panalytical, Malvern, UK) on ultrasonically dispersed suspensions (0.5 g/L) equilibrated across pH 2–8.

### 3.4. Cr(VI) Adsorption on ACSC@IONP

A 25 mg sample of ACSC@IONP or ACSC was added to 50 mL of K_2_Cr_2_O_7_ solution with the initial Cr(VI) concentration of 10 mg/L under neutral pH at 25 °C and stirred for 24 h. ACSs and ACSC were used as a comparison. After stirring, the residual Cr(VI) and Cr(total) concentrations were determined using diphenylcarbazide spectrophotometry and potassium permanganate oxidation-diphenylcarbazide spectrophotometry, respectively, according to Chinese national standards GB 7467-1987 and GB 7466-1987 [65,66]. The adsorption experiments were conducted in triplicate, and the error bars correspond to the standard deviation (SD).

Based on the characteristics of four ACSC@IONP samples, C@Fe-3 was selected for further adsorption research. A sample of 25 mg C@Fe-3 was added to 50 mL Cr(VI) solution of Cr(VI) initial concentration of 50 mg/L and pH 2. After stirring for 24 h, samples were taken for Cr(VI) determination. Then, the C@Fe-3 was separated, placed in 50 mL 1 mol/L NaOH solution, and stirred for 24 h for desorption. The desorbed C@Fe-3 was then reused as the adsorbent, and the adsorption–desorption cycle was repeated four times.

In the pH effect experiments, the pH of the Cr(VI) solution was adjusted to 1, 2, 3, 4, 5, 6, and 7 using HCl and NaOH, respectively. The removal of Cr(VI) with competitive ions (Cl^−^, NO_3_^−^, SO_4_^2−^) with molar ratios to Cr_2_O_7_^2−^ of 8, 16, 32, and 64 was conducted at pH 2. In the isothermal adsorption experiment, the adsorbent was investigated in Cr(VI) solutions with various concentrations of 20–100 mg/L at 30 °C, 40 °C, and 50 °C, respectively. In the kinetic experiment, 100 mg adsorbent was placed in 1000 mL of Cr(VI) solution (50 mg/L, pH 2). Samples were taken at different intervals to determine the Cr(VI) and Cr(total) concentrations.

## 4. Conclusions

In this work, a three-dimensional porous ACSC@IONP nanocomposite was successfully synthesized through a facile method involving synchronous pyrolysis carbonization and Fe^3+^ reduction. XRD, Raman, FTIR, SEM and TEM characterization confirmed the hierarchical porous structure of ACSC with uniformly dispersed Fe_3_O_4_ nanocrystals. The composite C@Fe-3 demonstrated exceptional Cr(VI) and Cr(total) removal efficiency at acidic pH < 2, driven by combined adsorption and reduction pathways. The reduction of Cr(VI) to Cr(III) and subsequent immobilization via precipitation or surface complexation ensured effective Cr(total) removal. Rapid kinetics equilibrium, high selectivity against interfering anions, and stable recyclability highlighted its practical potential. The Sips model further elucidated the heterogeneous energy distribution of adsorption sites. The effective anchoring of iron oxide nanoparticles on ACSC, after five adsorption–desorption cycles, the adsorption efficiency of Cr(VI) remained at 65%. This result proves that ACSC and IONP composites have good regeneration ability and recycling potential. By leveraging the innate hierarchical porosity of Artemia cyst shells, this work transcends the kinetic limitations of conventional Fe-biochars, achieving rapid total chromium management through 3D pore-engineered adsorption-reduction synergy. The structural innovation further advances the design of cost-effective, biochar-based nanocomposites for targeted heavy metal remediation, offering a sustainable solution to chromium pollution in complex aqueous environments.

## Figures and Tables

**Table 1:** Comparison of adsorption equilibrium time of chromium adsorbents.

Adsorbents	Equilibrium Time (min)	Initial Cr Concentration (mg/L)	Adsorbent Dose (g/L)	Ref.
Kiwifruit branch biochar (KBC)	510	-	0.09 g	[47]
Ball milling corn straw magnetic biochar (MBC)	80	100	0.5	[48]
AC-Fe_3_O_4_ nanocomposite	90	40	1.0	[49]
Cellulose magnetic biochar (DEBC)	180	15	0.5	[50]
Hickory ball milled biochar (BM-Fe-HC)	240	30	0.5	[51]
Magnetic powdered activated carbon (Mag-PAC)	720	300	3.0	[52]
FeCl_3_-modified lotus stem-based biochar(FeCl_3_@LS-BC)	900	10	1.6	[19]
Iron-clay biochar composite prepared from invasive *Populus nigra* (PFB)	1080	100	2.0	[33]
Rice husk magnetic biochar (MBC)	1120	100	1.0	[39]
Activated carbon/chitosan composite (AC/CS)	50	20	1.0	[53]
Corn straw biochar (Fe/N-PBC)	120	100	0.2	[54]
Ag/ZnO-AC nanocomposite	3600	20	0.4 g	[55]
Coating activated carbon with polysulfide rubber (AC-PSR)	5760	10	3.0	[56]
Three-dimensional porous *Artemia* cyst shell biochar-supported iron oxide nanoparticles (ACSC@INOP)	10 for Cr(VI) 5 for Cr(total)	50	0.1	This work

## Data Availability

Data presented in this article are available on request from the corresponding author.

## Supplementary Materials

The following supporting information can be downloaded at: https://www.mdpi.com/article/10.3390/molecules30081743/s1, Figure S1. EDS of C@Fe-3 before and after Cr adsorption; Figure S2. (a) N_2_ adsorption/desorption isotherms of ACSC and ACSC@IONP; (b) pore size distribution of the ACSC and ACSC@IONP by the Barrett–Joyner–Halenda (BJH) method; Figure S3. EDS of C@Fe-1 before Cr adsorption; Figure S4. Comparison of desorption rates of different desorption agents; Table S1. The relative element content of C@Fe-3 before and after Cr(VI) adsorption; Table S2. The regression coefficients of the removal kinetics models; Table S3. The regression coefficients of the removal kinetics models; Table S4. The regression coefficients of the isothermal adsorption models; Table S5. Iron leaching rate of C@Fe-3.
