# Supplementary material for: Three-Dimensional Porous Artemia Cyst Shell Biochar-Supported Iron Oxide Nanoparticles for Efficient Removal of Chromium from Wastewater
Source: Molecules. 2025 Apr 13;30(8):1743. doi: 10.3390/molecules30081743 (PMC12029975; doi:10.3390/molecules30081743)
Supplement: Supplementary file 1 [file molecules-30-01743-s001.zip › molecules-3530543-supplementary.pdf]

Supplementary Materials

Three-Dimensional Porous *Artemia* Cyst Shell Biochar-Supported Iron Oxide Nanoparticles for Efficient Removal of Chromium from Wastewater

Yu Gao, Ying Liu, Xu Zhao, Xinchao Liu, Qina Sun * and Tifeng Jiao *

State Key Laboratory of Metastable Materials Science and Technology, Hebei Key Laboratory of Heavy Metal Deep-Remediation in Water and Resource Reuse, School of Environmental and Chemical Engineering, Yanshan University, Qinhuangdao 066004, China

*Correspondence: sunqn@ysu.edu.cn (Q.S.); tfjiao@ysu.edu.cn (T.J.)

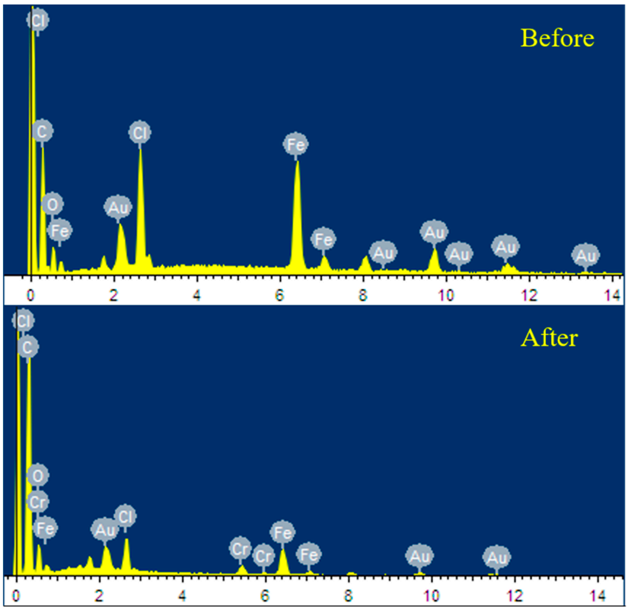

Figure S1. EDS of C@Fe-3 before and after Cr adsorption.

Table S1. The relative element content of C@Fe-3 before and after Cr(VI) adsorption.

Elements	Relative content (wt.%)	
	Before	After
C	54.2	68.5
O	10.9	16.3
Fe	21.2	6.7
Cr	0	1.7
Cl	7.0	2.5
Au*	6.7	4.3

*Au originates from the conductive coating applied during sample preparation for SEM-EDS analysis.

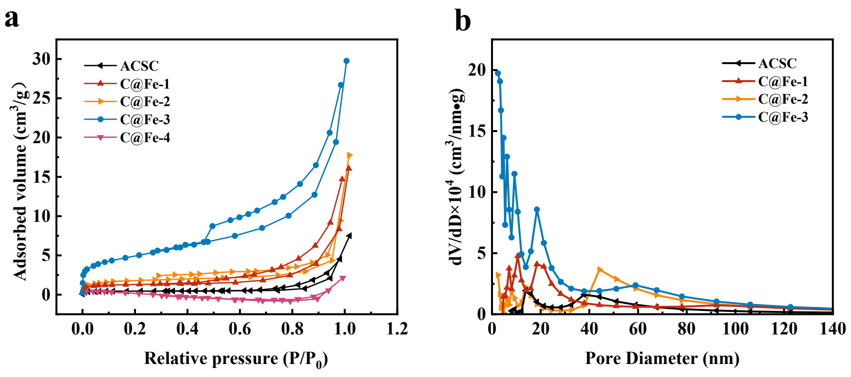

Figure S2. (a) N_2 adsorption/desorption isotherms of ACSC and ACSC@IONP; (b) pore size distribution of the ACSC and ACSC@IONP by the Barrett–Joyner–Halenda (BJH) method.

Table S2. Specific surface area and pore structure of ACSC and ACSC@IONP.

Materials	Specific surface area (m^2/g)	Volume (cm^3/g)	Average pore size (nm)
ACSC	1.6776	0.008311	19.817
C@Fe-1	4.8271	0.016535	13.702
C@Fe-2	6.7538	0.018970	11.235
C@Fe-3	17.657	0.039389	8.9233

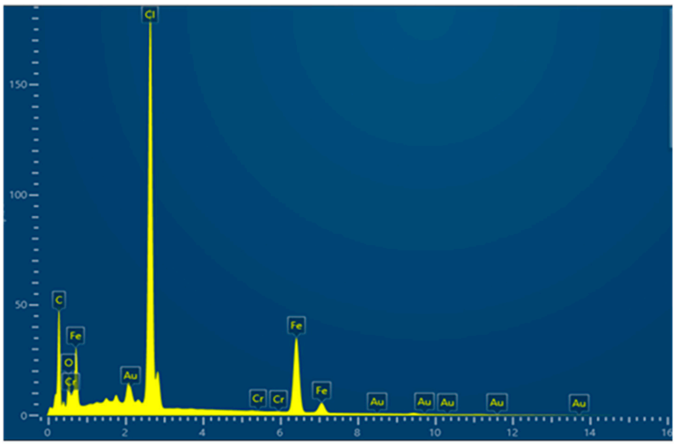

Figure S3. EDS of C@Fe-1 before Cr adsorption

Table S3. The regression coefficients of the removal kinetics models

	Pseudo-first-order model			Pseudo-second-order model		
	Q_e (mg/g)	K_1 (1/min)	R^2	Q_e (mg/g)	K_2 (g/(mg·min))	R^2
Cr(VI)	39.6	0.555	0.999	41.9	0.019	0.996
Cr(total)	21.1	1.753	0.998	21.3	0.223	0.997

Table S4. The regression coefficients of the isothermal adsorption models

Temperature (°C)		Langmuir			Freundlich			Sips			
		Q_{max} (mg/g)	b (L/mg)	R^2	K_F (mg/g)	n	R^2	Q_{max} (mg/g)	K_S (L/mg)	n_s	R^2
30	Cr(total)	74.58	0.22	0.91	42.78	8.74	0.66	71.60	0.18	1.37	0.93
	Cr(VI)	91.95	0.19	0.87	36.80	4.82	0.94	140.29	0.048	0.44	0.95
40	Cr(total)	121.96	0.03	0.83	33.95	3.49	0.71	94.85	0.14	2.68	0.87
	Cr(VI)	100.04	14.29	0.95	77.73	8.63	0.98	95.09	0.21	0.86	0.99
50	Cr(total)	143.28	0.10	0.88	33.89	2.95	0.76	110.09	0.13	2.15	0.95
	Cr(VI)	111.21	1.15	0.89	59.55	4.24	0.98	290.87	0.017	0.34	0.99

Commented [QS1]: Units in the table are change from the format "mg • g^{-1}" to mg/g.

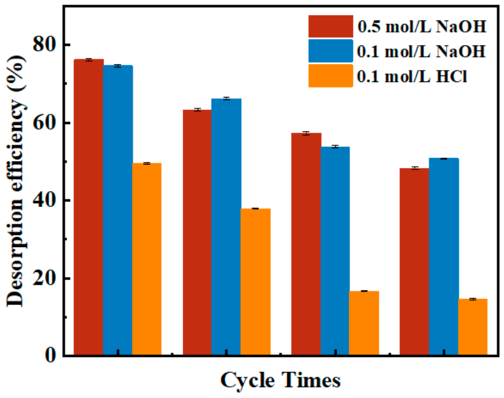

Figure S4. Comparison of desorption rates of different desorption agents

Table S5. Iron leaching rate of C@Fe-3

Materials	Weight (g)	Leaching rate (%)
C@Fe-3	0.025	1.71

Commented [M2]: Should this be Table S5? There is table S4 before

Commented [QS3R2]: It should be S5, and has been revised.
